# Comparison of Carbon Isotope Ratio Measurement of the Vanillin Methoxy Group by GC-IRMS and ^13^C-qNMR

**DOI:** 10.1021/jasms.3c00327

**Published:** 2023-11-28

**Authors:** Markus Greule, Phuong Mai Le, Juris Meija, Zoltán Mester, Frank Keppler

**Affiliations:** † Institute of Earth Sciences, Heidelberg University, Im Neuenheimer Feld 234-236, 69120 Heidelberg, Germany; ‡ Metrology, National Research Council Canada, 1200 Montreal Road, Ottawa, ON K1A 0R6, Canada

**Keywords:** Site-specific carbon isotope, vanillin methoxy group, GC-IRMS, ^13^C-qNMR

## Abstract

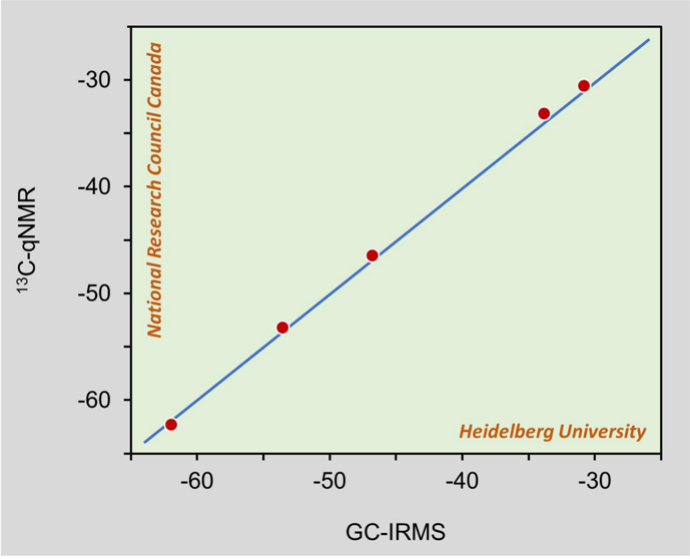

Site-specific carbon isotope ratio measurements by quantitative ^13^C NMR (^13^C-qNMR), Orbitrap-MS, and GC-IRMS offer a new dimension to conventional bulk carbon isotope ratio measurements used in food provenance, forensics, and a number of other applications. While the site-specific measurements of carbon isotope ratios in vanillin by ^13^C-qNMR or Orbitrap-MS are powerful new tools in food analysis, there are a limited number of studies regarding the validity of these measurement results. Here we present carbon site-specific measurements of vanillin by GC-IRMS and ^13^C-qNMR for methoxy carbon. Carbon isotope delta (δ^13^C) values obtained by these different measurement approaches demonstrate remarkable agreement; in five vanillin samples whose bulk δ^13^C values ranged from −31‰ to −26‰, their δ^13^C values of the methoxy carbon ranged from −62.4‰ to −30.6‰, yet the difference between the results of the two analytical approaches was within ±0.6‰. While the GC-IRMS approach afforded up to 9-fold lower uncertainties and required 100-fold less sample compared to the ^13^C-qNMR, the ^13^C-qNMR is able to assign δ^13^C values to all carbon atoms in the molecule, not just the cleavable methoxy group.

## Introduction

Stable isotope ratio analysis of elements has become a powerful analytical approach used in many scientific fields such as paleoclimatology, food authentication, forensics, plant ecology and atmospheric sciences.^1^ Bulk carbon isotope delta (δ^13^C, a common shorthand for δ(^13^C)) measurements are typically accomplished by using isotope ratio mass spectrometry (IRMS) or cavity ring down spectroscopy (CRDS). In contrast, ^13^C-qNMR is a powerful tool to measure carbon isotope ratios at individual positions (site-specific) within a molecule.^2^ The ^13^C-qNMR used in carbon isotope ratio measurement is also known as irm-^13^C NMR: isotope ratio measured by NMR, and as SNIF-NMR: Site-Specific Natural Isotope Fractionation determined by NMR.^2^ Recently, the high-resolution Orbitrap-MS was also used to measure site-specific carbon isotopic composition in serine.^3^ Thus, the ^13^C- qNMR and Orbitrap-MS direct access to site-specific isotope compositions of compounds at natural abundance provides much more information for classification and/or discrimination of molecules as a function of their geographical and/or chemical origin, especially in food products.^2,4,5^

However, applying IRMS for site-specific determination of carbon isotope ratios of the methoxy group in a given molecule is also possible, and this offers a means to validate the ^13^C-qNMR measurements. For this, one can employ the Zeisel method which cleaves methoxy groups from the target molecule by forming iodomethane (CH_3_I).^6−8^ This method has been demonstrated for a broad range of plant-based materials including wood and leaves,^9−12^ lignins or pectins,^13,14^ and specific molecules such as polygalacturonic acid or vanillin.^15,16^ In this context, plant methoxy groups have been shown to have distinct carbon isotopic patterns that are often significantly depleted in ^13^C relative to that of the bulk biomass or the whole molecule. Thus, measurements of the stable carbon isotopes of methoxy groups have great potential, for example, for paleoclimate studies,^12,17−20^ to determine the origin of coal bed methane^21,22^ or to study the freshness of fruits and vegetables,^23^ authenticity of vanillin^15,16,24^ and biomethylation processes.^25^ It is therefore important to establish the accuracy of the methoxy carbon isotope ratio measurements by using two independent analytical techniques on a well-characterized substance of high purity. We chose vanillin reference materials VANA-1 and VANB-1 from the National Research Council (NRC) for this purpose along with additional vanillin samples.

**Figure 1:**
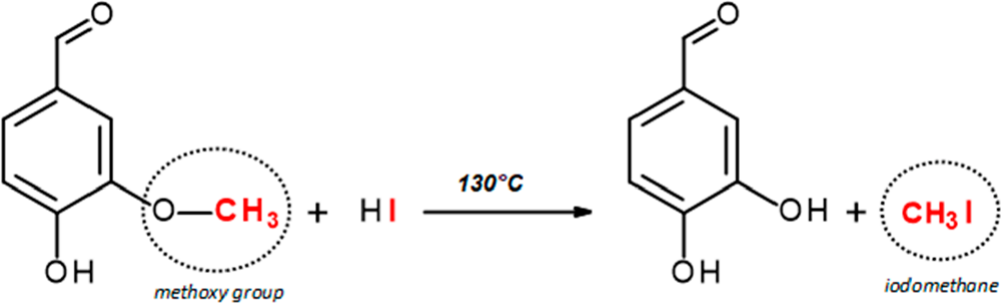
Zeisel reaction between vanillin and hydriodic acid (HI).

In this context, GC-IRMS analysis of methoxy groups is done by conversion to CH_3_I (Figure 1).^24^ Bulk δ^13^C values of vanillin materials show an overall range from −15‰ to −37‰ which can be broken down into three broad categories: natural vanillin (−15‰ to −22‰), biosynthetic vanillin (−19‰ to −37‰), and synthetic vanillin (−26‰ to −31‰).^15,16,24^ It is clear that the bulk δ^13^C values provide little distinction between these sources. In contrast, the δ^13^C values of the methoxy groups (δ^13^C_OCH3_) of the same vanillin samples show a significantly wider range from −7‰ to −53‰: natural vanillin (−7‰ to −26‰), biosynthetic vanillin (−23‰ to −50‰), and synthetic vanillin (−19‰ to −53‰).^15,16,24^ These larger differences in the δ^13^C_OCH3_ values offer better distinction between natural, biosynthetic, and synthetic vanillin samples. In addition, the combined information on δ^13^C values from bulk and methoxy groups makes it more difficult to counterfeit vanillin.

**Figure 2:**
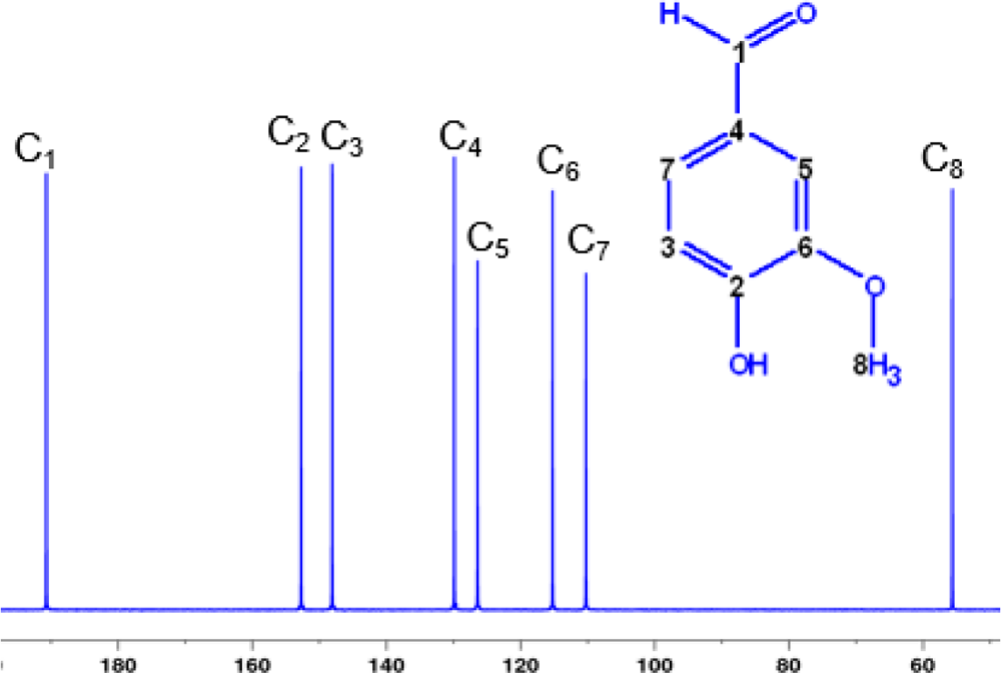
^13^C NMR spectrum of vanillin in acetone-*d*_6_ recorded on 400 MHz NMR. Numbering of carbon atoms is in the order of decreasing ^13^C chemical shift.

Furthermore, the carbon isotope ratio measurement by ^13^C-qNMR reveals all spectrally resolved sites in the compound and thus provides site-specific measurements (Figure 2). The isotopic ^13^C-qNMR method has been successfully applied and improved to authenticate the origin of vanillin and to elucidate the mechanisms of the bioconversion of ferulic acid to vanillin.^5,26−28^ Recently, this method was used for the certification of site-specific carbon isotope composition of the two vanillin certified reference materials, VANA-1 and VANB-1 from National Research Council of Canada.^4^ An interlaboratory comparison study conducted with the NMR laboratory of Nantes University (France), which also used the same ^13^C-qNMR method, showed consistent results of the carbon isotopic profile of VANA-1 and VANB-1.^4^ However, these studies did not employ independent methods to validate these results.

In this study, we compared ^13^C-qNMR and GC-IRMS methods for site-specific carbon isotope ratio measurements in five vanillin samples to validate these independent measurement approaches. The agreement between these analytical approaches would also demonstrate the applicability and commutability of these certified reference materials for both analytical approaches.

## Material and Methods

### Chemicals

Acetone-*d*_6_ was purchased from Cambridge Isotope Laboratories (Andover, MA, USA). Chromium acetylacetonate Cr(Acac)_3_, 99% and HPLC/Spectro grade acetone were obtained from Sigma-Aldrich (Oakville, ON, Canada). IRMS reference materials IAEA-CH-6, USGS65, IAEA-600, NBS22, USGS61, IAEA-603, IAEA-610, IAEA-611, and IAEA-612 were obtained from the International Atomic Energy Agency. NMR tubes (5 mm diameter) were obtained from Wilmad LabGlass (Buena, New Jersey, USA). Five vanillin materials, VANA-1, VANB-1 (NMR analysis in previous study^4^), VAN-1, VAN-4, and VAN-8 (NMR analysis in this study; GC-IRMS of all five vanillin samples in this study), are all synthetic materials of high chemical purity (≥99%) obtained from Fisher Scientific (Waltham, MA, USA), Sigma-Aldrich (St. Louis, MO, USA), Alfa Aesar (product of USA), and Sigma-Aldrich (product of China), respectively.

### NMR Analyses

#### Preparation of Vanillin Samples for ^13^C-qNMR Measurements

The preparation of vanillin samples followed the protocol described in Le et al.^4^ Briefly, approximately 250 mg of vanillin was weighed in a glass vial of 2 mL of 400 μL of acetone-*d*_6_ and 100 μL of Cr(Acac)_3_ solution (0.1 M) was added successively. The resulting solution was thoroughly mixed and then directly filtered into a 5 mm NMR tube.

#### Isotopic Composition of Vanillin Samples by ^13^C-qNMR

The details regarding the decoupling parameters, instrument calibration, and experimental setup for the site-specific carbon isotope measurements are described by Le et al.^4^ All NMR measurements were performed on a Bruker Avance III 400 MHz spectrometer equipped with a 5 mm Broad Band Observe ^1^H with the option of ^19^F decoupling (BBFO) probe, operated at 100.62 MHz. The temperature of the sample was set at 303 K. Probe ^13^C/^1^H tuning and matching were performed at the recording frequency of 100.62 MHz. Inverse gated adiabatic decoupling pulses were used for the ^1^H decoupling. The ^13^C NMR acquisition parameters were: sampling period, 0.7 s; spectral width, 220 ppm; 90°-^13^C pulse, 10.25 μs for most vanillin sample; repetition delay, 20 s (greater than ten times the longest *T*_1_ relaxation time); number of scans, 400 to reach an SNR of 700 on the carbon 5 at around 127 ppm. Five spectra were consecutively acquired for each sample. The carbon signal peak area integration was carried out with a numerical model implemented in R package Rnmrfit.^29^

### IRMS analyses

#### Bulk Isotope Delta Measurements of Vanillin

The bulk δ^13^C values of vanillin materials VANA-1, VANB-1 (previous study^4^), VAN-1, VAN-4, and VAN-8 (this study) were determined by elemental analysis (EA) combustion IRMS at the NRC. Details of the standard procedure for δ^13^C measurements are described by Chartrand et al.^30^ In brief, approximately 650 μg of vanillin samples and an appropriate amount of isotope reference materials were weighed into 5 × 3.5 mm tin capsules (Elemental Microanalysis; Okehampton, UK) and loaded onto an elemental analyzer (Vario EL III; Elementar Americas Inc., Mt. Laurel, NJ, USA) interfaced with a gas flow controller (Conflow III; Thermo Fisher; Bremen, Germany) to an isotope ratio mass spectrometer (Delta+XP; Thermo Fisher; Bremen, Germany). Combustion and reduction reactors were set to 950 and 500 °C, respectively. Helium dilution on the Conflow III was set to 0.5 bar (7 psi) pressure. All δ^13^C measurements were calibrated on the VPDB scale using nine reference materials (with their δ^13^C values and standard uncertainties given in the parentheses): IAEA-CH-6 (−10.45 ± 0.10‰), USGS65 (−20.29 ± 0.10‰), IAEA-600 (−27.77 ± 0.10‰), NBS22 (−30.03 ± 0.12‰), USGS61 (−35.05 ± 0.10‰), IAEA-603 (+2.474 ± 0.046‰), IAEA-610 (−9.145 ± 0.038‰), IAEA-611 (−30.925 ± 0.042‰), and IAEA-612 (−36.878 ± 0.052‰). The δ^13^C values used herein for these reference materials differ slightly from their certified values due to the discontinuity of the VPDB scale realizations which is discussed at great detail by Helie et al.^31^ Bulk values of δ^13^C for VANA-1, VANB-1, VAN-1, VAN-4, and VAN-8 relative to the VPDB are shown in Table 1.

**Table 1:** Bulk and Site-Specific δ^13^C Values in Five Vanillin Samples as Measured by ^13^C-qNMR and GC-IRMS (with the Associated 95% confidence Intervals, Uncertainty Coefficient *k* = 2, All Values in ‰)

laboratory	carbon atom	VANA-1^a^	VANB-1^a^	VAN-1	VAN-4	VAN-8
NRC	bulk	–31.30 ± 0.06	–25.85 ± 0.05	–29.81 ± 0.06	–31.10 ± 0.08	–26.19 ± 0.07
NRC	C1	–21.36 ± 1.40	–20.07 ± 1.40	–22.81 ± 1.40	–19.98 ± 1.40	–22.03 ± 1.40
NRC	C2	–32.09 ± 1.40	–29.87 ± 1.40	–32.94 ± 1.40	–28.88 ± 1.40	–29.92 ± 1.40
NRC	C3	–32.52 ± 1.40	–32.77 ± 1.40	–33.24 ± 1.40	–31.28 ± 1.40	–33.59 ± 1.40
NRC	C4	–28.26 ± 1.40	–24.48 ± 1.40	–31.64 ± 1.40	–26.83 ± 1.40	–28.96 ± 1.40
NRC	C5	–29.84 ± 1.40	–23.85 ± 1.40	–23.31 ± 1.40	–28.08 ± 1.40	–19.02 ± 1.40
NRC	C6	–29.38 ± 1.40	–26.38 ± 1.40	–27.53 ± 1.40	–26.80 ± 1.40	–24.02 ± 1.40
NRC	C7	–23.73 ± 1.40	–18.82 ± 1.40	–21.04 ± 1.40	–24.59 ± 1.40	–18.32 ± 1.40
NRC	C8 (NMR)	–53.25 ± 1.40	–30.59 ± 1.40	–46.53 ± 1.40	–62.36 ± 1.40	–33.20 ± 1.40
Heidelberg	C8 (IRMS)	–53.53 ± 0.26	–30.80 ± 0.16	–46.76 ± 0.27	–61.90 ± 0.29	–33.81 ± 0.41

a NRC measurements of VANA-1 and VANB-1 are from Le et al.^4^

#### Generation of Iodomethane from Vanillin for GC-IRMS Analysis

Analysis of δ^13^C values of CH_3_I, released upon treatment of the vanillin samples with 57% aqueous solution of hydriodic acid (Acros, Thermo Fisher Scientific, Geel, Belgium) was carried out using the method described by Greule et al.^7^ Hydriodic acid (0.25 mL) was added to the vanillin samples (2.5 mg) in a crimp-top glass vial (1.5 mL; IVA Analysentechnik, Meerbusch, Germany). The vials were sealed with crimp caps containing PTFE-lined butyl rubber septa (thickness 0.9 mm) and incubated for 30 min at 130 °C. After heating, the samples were allowed to equilibrate at room temperature (22 ± 0.5 °C) for at least 30 min before 30 μL of the headspace was directly injected into the GC using a 100 μL gastight syringe (SGE Analytical Science).

#### Carbon Isotope Analysis Using GC-C-IRMS

Carbon isotope analysis was performed using GC-combustion (C)-IRMS and the δ^13^C values of CH_3_I were measured using an HP 6890N gas chromatograph (Agilent, Santa Clara, USA) equipped with an auto sampler A200S (CTC Analytics, Zwingen, Switzerland), coupled to a MAT253 isotope ratio mass spectrometer (Thermo Fisher Scientific, Bremen, Germany) via an oxidation reactor [ceramic tube (Al_2_O_3_), length 320 mm, 0.5 mm i.d., with Cu/Ni/Pt wires inside (activated by oxygen), reactor temperature 960 °C] and a GC Combustion III Interface (ThermoQuest Finnigan, Bremen, Germany). The GC was fitted with a Zebron ZB-5MS capillary column (Phenomenex, Torrance, USA) (30 m × 0.25 mm i.d., df = 1 μm), and the following GC conditions were employed: split injection (10:1), initial oven temperature at 40 °C for 3.8 min, ramp at 50 °C/min to 110 °C. High-purity helium 5N (purity ≥ 99.999%) was used as carrier gas at a constant flow of 1.8 mL/min. A tank of high-purity carbon dioxide (99.995% or N45, Air Liquide, Düsseldorf, Germany) was used as the monitoring gas. The δ^13^C values were calibrated with linear regression^32^ using reference materials HUBG1 and HUBG2. These two standards are both methyl sulfates, which contain only one carbon atom in the molecule in the form of a methoxy group.^33^ They are therefore well suited as standard materials for the isotopic analysis of methoxy groups. Furthermore, these two reference material values span a relatively wide range on the VPDB scale covering most of the natural δ^13^C values of terrestrial plant methoxy groups.

Both materials HUBG1 and HUBG2 were measured by EA-IRMS and calibrated to the VPDB scale using the reference material IAEA-603 (+2.474 ± 0.05‰) and an in-house standard (acetanilide; −30.06 ± 0.20‰), which, in turn, was calibrated against the two reference materials NBS22 (−30.03 ± 0.08‰) and USGS44 (−42.21 ± 0.10‰).^34^ The calibrated δ^13^C values for HUBG1 and HUBG2 are −50.21 ± 0.08‰ (*N* = 14) and +1.61 ± 0.05‰ (*N* = 16), respectively.^33^ Here, all values following the “±” sign are expanded uncertainties (uncertainty coefficient *k* = 2) at 95% confidence level. It should be noted here that the values of HUBG1 and HUBG2 reported in Greule et al.^33^ differ slightly from the values used herein due to the small discontinuity in the realization of the VPDB scale.^31^ In this work, all δ^13^C values are expressed relative to the VPDB on the so-called VPDB2006 scale which has been set by adopting fixed values to reference materials NBS19 and LSVEC.^31^

#### NMR Data Processing and Report of Carbon Isotope Values

The site-specific carbon isotope ratios for each vanillin measured by ^13^C-qNMR and were calculated based on the bulk δ^13^C values obtained by IRMS and a ^13^C/^12^C ratio of R = 0.011108 for the VPDB, which is the weighted average of three recent independent estimates.^35−37^ In short, δ^13^C values for each carbon atom were then calculated from the processed NMR spectra using the areas of the site-specific carbon atoms corresponding to the central peaks observed in the ^13^C NMR spectra and the areas of the satellite peaks arising from the ^13^C–^13^C interactions as described in detail by Le et al.^4^

## Results and Discussion

### Comparison of GC-IRMS and ^13^C-qNMR Results

Bulk δ^13^C values of the five vanillin materials VANA-1, VANB-1, VAN-1, VAN-4, and VAN-8 were obtained by EA-IRMS with values ranging between −31.30‰ and −25.85‰ relative to the VPDB (see Table 1). Note that, in order to obtain the site-specific carbon isotope ratios by ^13^C-qNMR, one must scale them using the corresponding bulk δ^13^C values. The δ^13^C values of the methoxy groups (C8) differ significantly from all other carbon atoms and range from −62‰ to −31‰ (Table 1). This dispersion likely reflects the different synthetic routes and/or starting materials for these vanillin samples. Moreover, the isotopic composition of the carbon of the methoxy groups (C8) is strongly depleted in ^13^C for three vanillin samples (VANA-1, VAN-1, and VAN-4).

Although the isotopic compositions of carbon in the benzene ring of the five vanillin materials are somewhat similar, C7 carbon shows the highest δ^13^C values in all materials. In addition, we observe strong positive correlations between the δ^13^C values of C7 and C8, between C1 and C4, and between C5 and C7.

The methoxy group (C8) δ^13^C values in the five materials studied here ranges from −30.6‰ to −62.4‰ whereas the difference in these values between the two measurement methods ranges from +0.5‰ to −0.6‰ (Table 1). The uncertainty associated with the δ^13^C_OCH3_ values from 5 to 10 replicate measurements obtained by GC-IRMS ranges from 0.16‰ to 0.41‰. Uncertainty estimates for δ^13^C_OCH3_ values of vanillin measured by ^13^C-qNMR are ±1.40‰. For detailed description of the estimated uncertainties, we refer to the study by Le et al.^4^ Thus, the results obtained by the two independent analytical approaches are in excellent agreement. This is also noteworthy in light of the fact that both methods use different reference materials to calibrate the δ^13^C values to the VPDB scale.

### Effect of Vanillin Provenance on Its Isotopic Composition

Most synthetic vanillin is produced from the petrochemical precursor guaiacol.^38^ Vanillin from this source has significantly more negative δ^13^C_OCH3_ values than the natural vanillin, ranging from −19‰ to −53‰.^15,16,24^ A natural raw material that has long been used for the synthesis of vanillin is coniferous (soft) wood, whose lignin component is mainly composed of coniferyl alcohol.^39,40^ Coniferyl alcohol has a methoxy group that is not altered during conversion to vanillin and thus carries the isotopic signature of the trees during lignin synthesis. Recent studies have shown that the δ^13^C values of the lignin methoxy groups are in the same range (approximately −19‰ to −28‰)^18−20^ as the δ^13^C values of the bulk wood or cellulose extracted from it. Thus, δ^13^C_OCH3_ values might not be clearly distinguishable between wood-derived vanillin and vanillin which is extracted from vanilla beans with δ^13^C_OCH3_ values for the latter ranging from −7‰ to −26‰.^15,16,24^

Another commonly used route to commercial vanillin is the bioconversion of natural starting materials, such as rice and corn. Ferulic acid can be extracted from both and is then converted to vanillin by microorganisms. If the ferulic acid is derived from rice, the methoxy groups of the resulting vanillin have negative δ^13^C values in the range of −50‰. In contrast, the δ^13^C_OCH3_ values of vanillin from ferulic acid derived from corn are approximately −23‰ which is similar to that of authentic vanillin from vanilla beans or vanillin derived from lignin.^16^ Vanillin can also be formed biosynthetically from precursors such as glucose^41^ in which case the methoxy group is formed during the bioconversion. For such vanillin, the δ^13^C_OCH3_ value is approximately −15‰.^15^

All in all, this shows that the δ^13^C_OCH3_ values of vanillin are influenced by the precursor compounds and the (bio)synthetic pathways. But, a clear distinction between authentic, biosynthetic, and synthetic vanillin based on bulk δ^13^C values are not feasible, especially when vanillin samples of different sources are mixed. Therefore, the intramolecular distribution of δ^13^C values obtained by site-specific stable carbon isotope analysis might be applied to better assess the origin of vanillin. Moreover, the authenticity assessment of vanillin can be further improved by using hydrogen isotope analysis. This is also possible by ^2^H-qNMR for all hydrogen atoms of the vanillin molecule and by site-specific GC-IRMS for the hydrogen atoms of the methoxy group.

## Conclusions

Here we showed remarkably consistent values of the δ^13^C measurements in the methoxy group from five vanillin samples as measured by two independent methods, GC-IRMS and ^13^C-qNMR. The measurements of δ^13^C_OCH3_ using GC-IRMS afforded nearly an order of magnitude lower uncertainties and required considerably lower sample amounts compared to those by ^13^C-qNMR. While previous studies have shown that the δ^13^C values provided by ^13^C-qNMR are consistent with those obtained using GC-IRMS for ethanol samples of different origin,^42^ this study extends such a comparison to a more complex biomolecule (vanillin) as a further contribution toward validation of ^13^C-qNMR^4^ and GC-IRMS^7^ methods for site-specific carbon isotope measurements.
